# Emotional mimicry signals pain empathy as evidenced by facial electromyography

**DOI:** 10.1038/srep16988

**Published:** 2015-12-09

**Authors:** Ya-Bin Sun, Yu-Zheng Wang, Jin-Yan Wang, Fei Luo

**Affiliations:** 1 Key Laboratory of Mental Health, Institute of Psychology, Chinese Academy of Sciences, 100101, Beijing, China; 2 University of Chinese Academy of Sciences, 100049, Beijing, China

## Abstract

Facial mimicry has been suggested to be a behavioral index for emotional empathy. The present study is the first to investigate the link between facial muscle activity and empathy for pain by facial electromyographic (EMG) recording while observers watched videos depicting real-life painful events. Three types of visual stimulus were used: an intact painful scene and arm-only (needle injection) and face only (painful expression) scenes. Enhanced EMG activity of the corrugator supercilii (CS) and zygomaticus major (ZM) muscles was found when observers viewed others in pain, supporting a unique pain expression that is distinct from the expression of basic emotions. In the intact video stimulus condition, CS activity was correlated positively with the empathic concern score and ZM activity, suggesting facial mimicry mediated empathy for pain. Cluster analysis of facial EMG responses revealed markedly different patterns among stimulus types, including response category, ratio, and temporal dynamics, indicating greater ecological validity of the intact scene in eliciting pain empathy as compared with partial scenes. This study is the first to quantitatively describe pain empathy in terms of facial EMG data. It may provide important evidence for facial mimicry as a behavioral indicator of pain empathy.

Painful events happen in everyday life. Pain is not only an intrapersonal experience, as generally thought, but also an interpersonal phenomenon that can affect observers^1^,^2^. Perceiving and understanding others’ suffering, commonly referred to as empathy for pain, automatically elevates physiological arousal (e.g., skin conductance)^3^, activates brain regions involved in the direct experience of pain^4^,^5^,^6^,^7^,^8^, and can make people feel pain as if they had received the noxious stimulation^9^. Empathy is defined as an emotional response to the observed experiences of another being, or the ability to share another’s feelings^10^. Great advances have been made in our understanding of empathy for pain, mainly by neuroimaging studies, which have revealed the shared representation between first-hand and vicarious pain experiences^11^,^12^. Previous studies have assessed pain empathy using brain-imaging techniques and self-report measures, given the lack of objective indicators for this daily social occurrence.

Previous research on embodied emotion has demonstrated that people react to others’ emotions with similar emotions^13^,^14^; mainly showing as they spontaneously mimic facial expressions when viewing others’ emotional faces. Emotional mimicry has been considered a special subset of behavioral mimicry^15^, which is defined as an automatic, matched motor response to another person’s behavior^13^. The mechanisms underlying this link between perception and behavior involve a shared representation network, i.e., activation of overlapping brain areas during both motor action and action observation^16^,^17^. Mimicry may help people to empathize with another person, thus understanding their emotional states^18^,^19^,^20^. It has been found that mimicking behavior is linked closely to the empathy trait, i.e., individuals with high empathy levels are more facially reactive^21^,^22^. The blocking of mimicking behavior interferes with emotion recognition and empathic responses^23^. Using transcranial magnetic stimulation, Avenanti *et al.* found a reduction of excitability of hand muscles during the mere observation of another person being pricked, providing solid evidence for the role that the motor system plays in empathy for pain^8^. Furthermore, associations have been reported between empathy deficit disorders, such as autism, and impairment of automatic mimicry^24^,^25^. For example, Minio-Paluello *et al.* revealed reduced empathic abilities in people with Asperger Syndrome (AS) by showing that participants with AS did not exhibit embodied empathic pain resonance when viewing others in pain^26^. Particularly, emotional mimicry paves the way of empathy^17^. Emotional mimicry has typically been assessed by exposing participants to visual stimuli, either static pictures or dynamic videos, while measuring the activity of specific facial muscles with electromyography (EMG)^27^ or the Facial Action Coding System (FACS)^28^. Corrugator supercilii (CS) activity (frowning) is proved to be a robust reaction to negative emotions, such as anger and sadness, and zygomaticus major (ZM) activity (smiling) indicates the presence of a smile face in response to happy emotions, according to numerous studies^13^,^27^,^29^,^30^. Thus, facial mimicking may indicate the occurrence of empathy in social interactions.

Although observers’ facial responses are important in conveying an understanding of perceivers, and their assessment can complement other measures such as neuroimaging, responses related to empathy for pain have not been investigated thoroughly. Despite no specific pain expression, existing evidence has supported that the facial expression of pain is distinct from the expression of basic emotions^31^. Typically, facial actions such as brow lowering (CS activity), cheek raising and lid tightening (orbicularis oculi activity), nose wrinkling and upper lip raising (levator labii activity), and eye closing are considered to comprise the “core” expression of pain^32^,^33^. An early study showed that observers’ orbicularis oculi reacted more strongly to painful than to neutral expressions, indicating the occurrence of pain-like facial behavior when seeing others in pain^34^. Lamm *et al.* also found that observers adopting self-perspectives showed enhanced orbicularis oculi activity in reaction to videos of patients undergoing painful sonar treatment^35^. Moreover, a recent study revealed that CS and orbicularis oculi activity increased with observers’ sense of responsibility for others’ suffering^36^. However, evidence for whether observers mimic the emotional expressions of persons in pain is conflicting. Reicherts *et al.* found no difference in observers’ facial muscle activity when viewing painful and neutral expressions^37^. Similarly, Mailhot *et al.* reported no specific facial response to the viewing of painful faces when exploring the priming effect of empathic processes on observers’ self-pain^38^. Thus, it remains unclear whether people mimic others’ painful facial expression when facing them in pain.

The contradictory results may be due to that pain is different from basic emotions (e.g., happiness and sadness, which are robust in causing facial mimicry^13^) in that it is often caused by an actual noxious stimulation, and may be underrepresented by facial expression only. Thus, intact scenes, i.e., those that depict cause (bodily injury) and effect (painful expression), may be better stimuli for the study of pain empathy. Besides, it’s common to see that people often wince when witness another individual in pain in daily life, which suggests the use of real-life pain situations may be especially effective. To our knowledge, two categories of stimulus are often used in pain empathy studies: injured body parts and facial expressions of pain^5^,^6^,^39^,^40^,^41^,^42^, and very few studies of empathy have involved the presentation of whole-body scenes to observers^9^, and no study has compared the effects of such scenes with those of injured body part and painful expression stimuli in eliciting empathic responses.

Accordingly, in order to explore whether the empathic response to another’s suffering involve emotional mimicry, a group of participants (group A, see supplementary table S1 and Fig. 1) were asked to watch intact real-life scenarios (pain vs. no-pain, depicting people receiving needle penetration or q-tip brushing, respectively) with their facial muscle activity synchronously measured by electromyography. To compare facial reaction elicited by different empathy-eliciting stimuli, another group of participants (group B, see supplementary table S1 and Fig. 1) were recruited and they were asked to watch videos depicting either a facial expression alone or an arm alone derived from the intact scenario. We hypothesized (a) that observers would exhibit enhanced CS activity and suppressed ZM activity in response to painful (*vs*. non-painful) scenarios for all three stimuli, and (b) that intact scenarios would elicit empathic reactions more effectively (including higher subjective ratings and more CS activity) than would traditionally used injured body scenarios. Our results demonstrate that observers show a facial expression of pain when facing others in pain, and the mimicking behavior correlate positively with their empathy trait. The phenomenon is more robust when intact scenarios are employed. We also quantitatively measure the proportion of observers showed facial mimicking behavior by a cluster analysis. These results confirm that emotional mimicry can be a behavior indicator of pain empathy.

## Results

### General information on targets and observers

Fifty-one participants (15 men, 36 women) received needle injections while being video recorded. Based on self-reported ratings and facial pain expressions and after matching on the basis of demographic characteristics (gender, age, education, and attractiveness), 20 of 51 video clips from 8 men and 12 women were selected as target stimuli for the formal experiment. Demographics and self-reported ratings of target subjects are presented in Supplementary Table S1. A significant difference in self-reported pain intensity was found between targets showing painful and neutral expressions (6.01 ± 2.38 *vs*. 2.88 ± 1.88, *t*[18] = 3.27, *P* = .004). No difference in pain-related unpleasantness was observed (3.98 ± 2.69 vs. 2.40 ± 2.00, *t*[18] = 1.49, *P* = .154), suggesting that the injection procedure did not cause obvious emotional distress.

Five of 69 observer participants were excluded due to insufficient numbers of trials after artifact rejection. For the remaining 64 participants, the percentage of excluded trials was 5.75%. Table 1 shows the demographic characteristics and trait empathy scores of observers. Gender, age, and education were comparable in the two groups. No significant difference in emotional empathy trait was observed between groups (*P* = .429 and *P* = .867, for C-IRI EC and PD score respectively).

### Observers’ empathic reactions to intact painful scenarios

Participants in group A (intact scene stimuli) reported significantly higher pain intensity and unpleasantness ratings for pain videos than for no-pain videos (*t*[29] = 16.67, *P* < .00001, *R*^2^ = .91 and *t*[29] = 12.00, *P* < .00001, *R*^2^ = .83, respectively; Table 2). Figure 2a shows changes in physiological measurements when participants watched the videos. More CS activity was observed in response to pain than to no-pain videos (*t*[29] = 3.37, *P* = .002, *R*^2^ = .28), suggesting that participants frowned more while watching painful scenes. Unexpectedly, participants also exhibited greater ZM activity during exposure to pain videos compared with no-pain videos (*t*[29] = 2.84, *P* = .008, *R*^2^ = .22). No significant difference was found in pulse rates elicited by the two video types (*t*[29] = .80, *P* = .432, *R*^2^ = .02). Figure 2b provides examples of pain video–induced facial EMG responses from two observers.

### Different empathic reactions to needle penetrated arms and pain expressions

For Group B, two-way ANOVA (condition: pain vs. no-pain; stimulus category: arm-only vs. face-only) of intensity and unpleasant response ratings revealed significant main effects of condition (*F*[1,33] = 265.65, *P* < .00001, *η*_*P*_^2^ = .89 and *F*[1,33] = 208.16, *P* < .00001, *η*_*P*_^2^ = .86, respectively) and stimulus category (*F*[1,33] = 12.68, *P* = .001, *η*_*P*_^2^ = .28 and *F*[1,33] = 30.54, *P* < .00001, *η*_*P*_^2^ = .48, respectively; Table 2). No significant condition × stimulus category interaction was identified for either rating (intensity: *F*[1,33] = 1.09, *P* = .305, *η*_*P*_^2^ = .03; unpleasantness: *F*[1,33] = .07, *P* = .798, *η*_*P*_^2^ = .002), suggesting that the effect of pain videos on observers’ emotional experience did not differ between arm-only and face-only visual stimuli. *Post hoc* results revealed more-unpleasant responses to arm-only than to face-only pain videos (*P* < .00001), although no difference in pain intensity ratings was observed (*P* = .118).

Significant main effects of condition (pain *vs*. no pain) were found on facial EMG data (CS: *F*[1,33] = 13.45, *P *= .0009, *η*_*P*_^2^ = .29; ZM: *F*[1,33] = 4.66, *P* < .038, *η*_*P*_^2^ = .12) and pulse rate (*F*[1,33] = 6.01, *P* < .020, *η*_*P*_^2^ = 0.15; Fig. 2a). The main effect of stimulus category (arm only *vs*. face only) was significant only for ZM activity (*F*[1,33] = 5.42, *P* < .026, *η*_*P*_^2^ = .14). No other significant effect was observed.

### Different empathic reactions to intact painful scenarios and needle penetrated arms

In between-subject comparisons, we highlighted the difference in induced responses between intact and arm-only (injured body part) scenes, as the latter are used most commonly in pain empathy research^6^,^11^,^39^. For pain intensity and unpleasant response ratings, ANOVA revealed significant main effects of condition (pain *vs*. no-pain; intensity: *F*[1,62] = 375.25, *P  *< .00001, *η*_*P*_^2^ = .86; unpleasantness: *F*[1,62] = 300.88, *P* < .00001, *η*_*P*_^2^ = .83) and group (intact *vs*. arm only; intensity: *F*[1,62] = 10.57, *P = *.002, *η*_*P*_^2^ = .15; unpleasantness: *F*[1,62] = 9.53, *P* = .003, *η*_*P*_^2^ = .13). No significant condition × group interaction was observed (Supplementary Table S2). *Post hoc* results revealed no significant difference between intact pain and arm-only pain ratings. For physiological measures, ANOVA showed significant main effects of pain on CS (*F*[1,62] = 15.23, *P *= .0002, *η*_*P*_^2^ = .20) and ZM activity (*F*[1,62] = 9.90, *P  *= .006, *η*_*P*_^2^ = .14) and on pulse rate (*F*[1,62] = 5.24, *P  *< .025, *η*_*P*_^2^ = .08), and a significant condition × group interaction for ZM responses (*F*[1,62] = 8.19, *P  *= .005, *η*_*P*_^2^ = .12). No other significant effect was observed (Supplementary Table S2).

### Correlation analysis

Pearson correlation analysis revealed significant correlations only in group A. The EC subscale score was correlated positively with CS activity (*r* = .44, *P* = .014; Fig. 2c), suggesting that those with greater self- reported empathy were prone to frown when witnessing others in pain. Interestingly, a significant but weak positive correlation was observed between CS and ZM activity (*r* = .38, *P* = .038), suggesting that both facial muscles may respond to painful visual stimuli in an interrelated manner. By contrast, no significant correlations were found in group B. There were no other correlations for both groups.

### Temporal dynamics of CS and ZM EMG activity

Cluster analysis revealed distinct CS activity patterns in response to the three stimulus types (Fig. 3a). The intact-scene stimuli elicited long-lasting EMG responses, whereas responses to arm-only and face-only videos were more scattered. Moreover, intact-scene videos elicited exclusively excitatory responses, whereas arm-only and face-only videos evoked some inhibition in addition to excitation, suggesting that presentation of a partial scene of a painful situation leads to CS relaxation in a portion of observers. Importantly, the ratio of excitation induced by arm-only pain videos did not differ from that evoked by intact pain videos (44% vs. 43%, Yates’ *χ*^2^[2]* = *3.25*, P* = .197 ), indicating that the stimuli elicited similar degrees of activation. The percentages of observers showing excitatory and inhibitory responses are shown in Supplementary Table S3.

The time course of ZM activity showed a striking difference between the intact-scene and partial-scene induced responses. As shown in Fig. 3b, in contrast to the robust responses elicited by intact-scene videos (i.e., 50 percent of observers showing excitatory or inhibitory responses), no significant responses can be seen under the partial-scene condition. The percentages of excitatory and inhibitory responses are illustrated in Supplementary Table S3.

## Discussion

The present study focused on observers’ empathic reactions to video stimuli depicting others in pain (i.e., receiving forearm needle injections), with synchronous facial EMG recording. Using within-subject and between-subject designs, we compared empathic responses (including subjective ratings and facial EMG signals) elicited by different stimuli (i.e., intact scene, arm-only and face-only videos) under pain and no-pain conditions. The study produced three major findings. First, regardless of stimulus type, pain videos induced significantly higher ratings (including pain intensity and unpleasant reaction) and greater facial EMG responses (including CS and ZM activity) than did no-pain videos. Second, the CS activity induced by intact scene viewing was correlated positively with observers’ EC subscale scores and ZM activity. Finally, cluster analysis of the temporal dynamics of facial EMG responses showed distinct patterns in response to the three video stimulus types.

The materials used to elicit empathy in the current study were video clips presenting real-life painful situations in which individuals received forearm needle injections. We evaluated the effectiveness of video stimulus types (whole-body scene, arm [injured body part] only, and face only), considering the whole-body painful scenario to have greater ecological validity because of its integrity and naturalness in depicting real-world events^43^. Intradermal needle injection is a common painful event that almost everyone has experienced in clinical settings. Thus, it can be expected to readily elicit empathic responses in observers while causing little harm to target subjects. As evidenced by our results, needle penetration did not cause some unpleasant experiences (the mean unpleasantness rating was <3).

Our results showed that observers’ ratings of others’ pain intensity and their own unpleasant feelings were consistently higher for painful than for non-painful scenes, regardless of stimulus type. These findings validate the effectiveness of the video stimuli. Within-subject comparison of group B revealed that more unpleasant responses were induced by watching forearm needle penetration than by viewing painful facial expressions. Observers may imagine themselves in pain when viewing scenes of injured body parts, generating feelings similar to those of the perceivers. By contrast, as humans rely largely on faces to identify others, observers would naturally not implicate themselves when seeing only others’ faces. In previous studies, participants reported more painful responses when they imagined themselves in pain than when they imagined others in pain^39^,^44^. In the present study, between-subject comparison showed no difference in ratings between intact and arm-only painful videos, indicating that whole-body and local-injury views have equal strength in eliciting empathic responses.

An important finding of the current study was that pain videos elicited a global increase in facial (CS and ZM) EMG activity compared with no-pain videos. Studies of basic emotion have demonstrated that CS activity is associated with negative emotions like fear and sadness, whereas ZM activity is considered to be a sign of positive emotion^28^,^45^. Even in the context of social interaction, increased CS and ZM activity seems to reflect valence-specific affective processing (for negative and positive emotions, respectively)^27^,^29^,^30^. We thus predicted that video stimuli depicting painful situations would enhance CS activity while suppressing ZM activity. This hypothesis was only partially supported. As expected, painful scenarios stimulated CS activity, suggested that observers experienced negative emotion when viewing others in pain. This result supports previous findings^35^,^36^. Furthermore, data from intact-scene painful video viewing revealed that CS activity was correlated positively with EC score, indicative of an empathic response. According to Davis^46^, the EC subscale measures other-oriented emotional empathy, or the tendency to experience sympathetic feelings such as warmth and compassion for others. Therefore, the positive correlation between CS EMG and EC subscale indicates that the specific muscle activity is indicative of an empathic response.

However, contrary to expectation, we also observed increased ZM activity in response to painful scenes. More interestingly, we found that ZM and CS activity were positively correlated. These results suggest that ZM activation plays a role in the generation of a painful facial expression. Normally, CS and ZM activity are incompatible in the expression of basic emotions^28^. The CS is considered a sign of negative emotion, while the ZM commonly occurs in a smile^47^. However, several investigations have provided evidence that pain expression can involve the activation of ZM muscle^31^,^33^,^48^,^49^. Craig and Patrick reported that lip corner pull, the action of ZM muscle, occurred during the experience of pain^49^. In their study, facial reactions to cold-pressor pain were evaluated based on FACS. They identified lip corner pull as a component of facial pain expression, because this facial movement was observed in 45 of the 72 subjects. In another study, Patrick *et al.* examined whether there was an overlap between electric-shock vs. cold-pressor induced facial actions, and found that the lip corner pull occurred only in cold-pressor pain but not in shock-related pain^48^. A recent study compared facial expressions of pain and basic emotions and showed some overlap of prototypical action units between these different emotions^31^. Lip corner pull occurred in 8 of 8 actors with happiness expression and 3 of 8 actors with pain expression. Likewise, Prkachin and Solomon analyzed facial expressions of people with shoulder pain, and found a medium effect size of pain on oblique lip pulling action (η^2^ = .15)^33^. According to the facial mimicry theory, observers spontaneously match others’ facial expressions of emotion^23^,^29^,^50^,^51^. From this point of view, the activation of both facial muscles when viewing others in pain may reflect observers’ mimicking of target subjects’ painful expressions. Previous neuroimaging studies have consistently demonstrated that observing others being injured or expressing pain automatically activates parts of neural circuits underlying first-hand pain experience, known as shared representation^1^,^7^,^11^,^12^,^52^. The human mirror neuron system has been shown to be closely related to empathy and facial mimicry^14^,^42^,^53^,^54^. However, as we did not measure facial EMG responses of target subjects, we cannot verify the congruence of physiological responses between target subjects and observers, and the evidence supporting facial mimicry theory was thus indirect.

Within-subject and between-subject comparisons failed to find a difference in the magnitude of facial EMG responses to different pain videos, suggesting that these stimuli produced similar effects on facial reactions. However, the time course of CS and ZM activity showed different patterns in response to whole-body and local-injury videos. Intact-scene video stimuli elicited exclusively excitatory responses in CS muscle, whereas arm-only and face-only videos produced excitatory and inhibitory CS activity. Intact-scene videos also induced longer and more continuous CS excitation as compared to partial-scene videos. Even more striking was the difference between intact-scene vs. partial-scene induced ZM activity, with 50 percent of observers showing significant response under the former condition in sharp contrast to the 0 percent under the latter condition. These findings may indicate greater effectiveness and ecological validity relative to body-part scenarios as empathy-eliciting material.

Cardiac response has been considered to be indicative of emotional responses. Although we simultaneously monitored subjects’ facial EMGs and heart rate during their exposure of visual stimuli, we did not find a correlation between CS or ZM activation and the changes in cardiac activity, and neither a relationship between these physiological measures (EMG or pulse rate) and subjective reports of emotional experience. Facial EMG has been thought to provide a direct measure of automatic mimicking, which changes in a fast and dynamic pattern. In contrast, heart-rate response is often slower to sympathetic activation^55^, and may reflect the allocation of attentional resources to salient stimuli instead of an emotional response. In addition, facial mimicry is an automatic and unconscious reaction, while heart rate response is generally associated with perception of emotional stimuli^56^. Especially for subjective report, it is under conscious control and is more likely to be discounted than nonverbal behavior when they are inconsistent^49^.

Some limitations of this study should be addressed. First, the durations of pain (15 s) and no-pain (7 s) videos were not matched. The full-length scenes of painful situations were important to depict the time-varying painful facial expressions of the target subjects. In contrast, 15 s of repeated skin brushing with a Q-tip (accompanied by an unchanged neutral facial expression) seemed too long for observers, who tired of watching the video and exhibited involuntary facial and body activity. Second, we did not use pre-injection baseline data from target subjects as a control. In other words, painful and non-painful scenarios were recorded with different participants. The reason for this approach was that subjects who showed apparent pain during needle injection also tended to be nervous in the baseline period, rendering maintenance of a steady, neutral expression difficult.

In summary, with the use of naturalistic videos depicting painful and non-painful situations as stimuli, the present study confirmed previous findings that observers showed overt signs of empathy, including increased pain ratings and activation of facial muscles, when viewing others in pain. In particular, simultaneous CS and ZM activation, which typically reflect negative and positive emotions, respectively, may indicate a characteristic feature of pain expression. More importantly, the correlation between empathy scores and facial EMG responses was observed only for whole-body painful scenarios, not for videos depicting local bodily injury or painful facial expressions, demonstrating the greater ecological validity of the intact scene in eliciting pain empathy. Considering the complexity of intact, natural scenes depicting real-life events, future studies may use eye tracking to capture the attention of observers when using this type of stimulus. The combined use of EMG recording and eye tracking may help to reveal the relationship between empathic responses and visual attention, and enhance our understanding of how empathy occurs in a social interaction context.

## Methods

### Participants

One hundred and seventy subjects participated in the present study. Fifty-one subjects (15 men, 36 women; mean age 21.57 ± 2.17 years) were recruited to produce empathy-eliciting videos, in which they served as empathy targets and received painful stimuli. Another 50 subjects (24 men, 26 women; mean age 23.04 ± 2.06 years) were recruited to rate the emotional valence and attractiveness of faces depicted in the videos. The remaining 69 subjects (23 men, 46 women; mean age 21.05 ± 2.02 years) participated in the formal experiment. All subjects were Chinese graduate or undergraduate students from neighboring universities. They had normal or corrected-to-normal vision, and no current or prior history of chronic pain or neurological or psychiatric disorder (self-reported). All participants provided written informed consent prior to participation and received monetary rewards after the experiment. The study was approved by the Research Ethics Committee of the Institute of Psychology, Chinese Academy of Sciences.

### Video stimuli

Video clips in which each individual received a painful needle prick were recorded and edited. Prior to recording, subjects were asked to wear light-colored clothing and no face makeup, and to push their hair back from the forehead. People with histories of cardiac disease or needle phobia were excluded for health reasons. Each subject received a .1-ml intradermal injection of normal saline (NS) in the left forearm through a 27-gauge needle over 15 s. Video clips were shot from a frontal view using a digital color camcorder (LEGRIA HF R18; Canon Inc., Tokyo, Japan), with subjects seated 1.5 m away with the left arm extended and held in the right hand. Subjects were instructed to attend to the injection and express their emotions through facial responses. The whole recording process lasted about 1 min, including the periods of pre-injection baseline, skin disinfection with a Q-tip, and NS injection. After the termination of recording, subjects were required to evaluate the intensity and unpleasantness of their pain experience on a visual analogue scale, with 0 indicating “no pain” and 10 indicating the “worst pain imaginable.”

A total of 51 video clips (intact version, 1440 × 1080 pixels) were produced. Due to individual differences in pain sensitivity and reactivity, only 19 of 51 subjects (seven men) displayed clear facial expressions of pain (according to the “core” expression of pain concept^33^,^57^; the remaining 32 subjects had neutral expressions during the injection. The video clips were thus divided into two sets reflecting pain (*n* = 19) and no pain (*n* = 32), depicting subjects being injected and being touched by a Q-tip, respectively. The video clips were edited using Windows Movie Maker 12 (Microsoft Inc., Seattle, WA, USA) and trimmed to desired length (15 s for pain videos, 7 s for no-pain videos). The pain and no-pain videos were further separated into two groups of smaller, time-paralleling clips (854 × 480 pixels each) depicting only faces and injected or Q-tip–touched forearms, respectively, using crop tools in the Leawo video converter software (Leawo Inc., Shenzhen, Guangdong, China).

Using a seven-point Likert scale ranging from 1 (very unattractive) to 7 (very attractive), 50 participants rated the attractiveness of each target face after viewing face-only videos. After matching based on age, gender, years of education, and facial attractiveness, 10 pain and 10 no-pain videos (each set depicting four men and six women) were selected as stimuli for the formal experiment (Supplementary Table S1). Eight of the 20 arm-only clips (four pain, four no pain) were used as stimuli because arms are much less recognizable than faces, and repeated presentation may cause habituation. Thus, the final set of video clips comprised intact and face-only clips from 20 target subjects, and arm-only clips from eight target subjects (see supplementary videos).

### Experimental design

**Figure 1:**
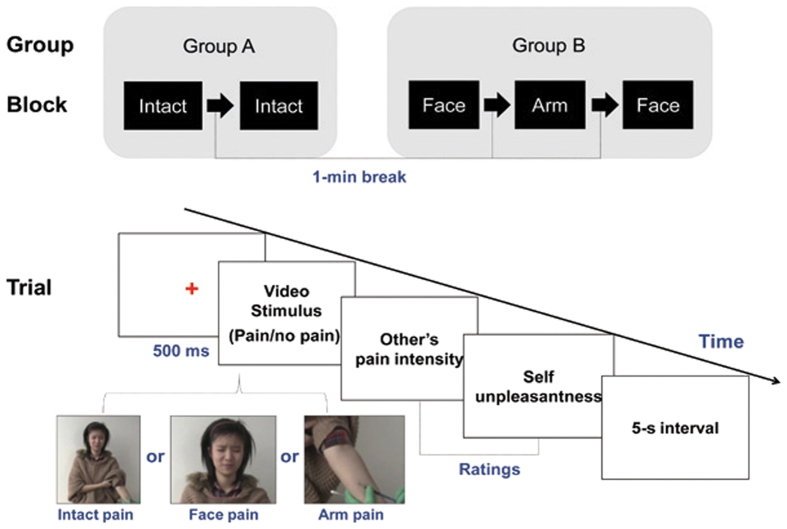
Experimental design and trial procedure. The study consisted of two conditions (pain and no pain) and three stimulus types (intact scene, arm only and face only), and involved a mixture of between-subject (group × condition) and within-subject (condition × stimulus type) analyses. Painful video stimuli are exemplified by screenshots (at the bottom of the figure) taken from video clips produced for this study.

**Figure 2:**
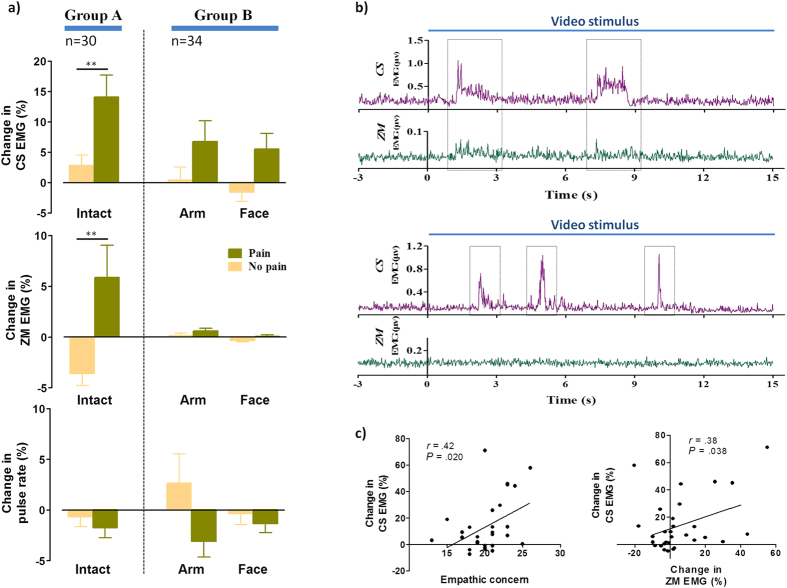
Physiological responses to video stimuli and relationship between facial electromyographic (EMG) data and empathy scores. (**a**) Within-subject and between-subject comparisons of physiological indices. Corrugator supercilii (CS) and zygomaticus major (ZM) EMG responses and pulse rate were converted to percent changes from baseline. Pain videos enhanced both facial EMG responses as compared with no-pain videos. **P < .01. Error bars represent standard errors of the mean. (**b**) Examples of facial EMG responses from two observers. Different spatial patterns and temporal dynamics can be seen in response to the same pain video clip, with activation of both facial muscles in one observer (upper) and CS activation only in the other observer (lower). Dotted outlines indicate the enhancement of EMG signals. (**c**) Positive correlations of CS EMG activity with empathic concern subscale score (left), and ZM EMG activity (right). Correlations were found only in trials employing the whole-body pain video, not in those using the arm-only and face-only pain videos.

**Figure 3:**
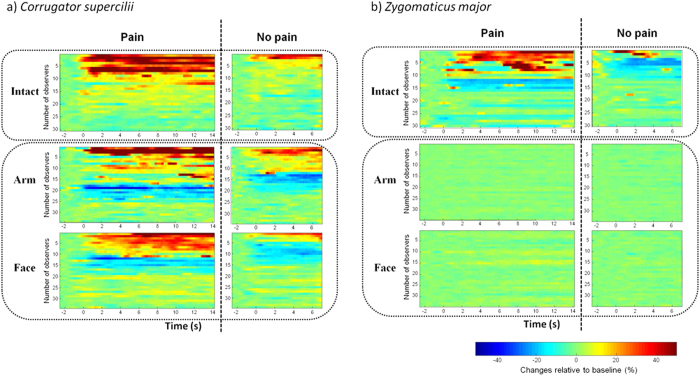
Temporal distribution patterns of corrugator supercilii (a) and zygomaticus major (b) electromyographic signals elicited by three types of video stimulus. Raw signals were transformed into percent changes from baseline. Participants with > 20% change were considered to be significantly excited or inhibited. Each line represents normalized activity of one participant. (**a**) The time course of CS activity showed different patterns in response to whole-body and local-injury videos. Intact-scene video stimuli elicited exclusively excitatory responses, whereas arm-only and face-only videos produced excitatory and inhibitory CS activity. Intact-scene videos also induced longer and more continuous CS excitation as compared to partial-scene videos. (**b**) The time course of ZM activity showed significant activation and inhibition in response to intact-scene videos. In contrast, however, neither activation nor inhibition was observed in response to arm-only and face-only videos.

Sixty-nine subjects were randomly assigned to groups A (intact scene stimuli) and B (face-only and arm-only stimuli). The whole procedure is illustrated in Fig. 1. Participants were seated in a sound-attenuated and temperature-controlled (22–24°C) room. After providing informed consent, they were given instructions for the task. Physiological signals were measured using Biopac MP150 equipment (Biopac Systems Inc., Santa Barbara, CA, USA). Electrodes were attached after cleaning the sites (face muscles and fingers) with alcohol swabs. Participants were asked to minimize body movement during the experiment. Task instructions and video stimuli were presented on a projection screen (60 × 70 cm) located approximately 2 m in front of participants. Stimulus generation and presentation, and response recording, were controlled by a personal computer using E-prime software (version 2.0; Psychology Tools Inc., Pittsburgh, PA, USA).

Participants in group A performed two blocks of 10 trials each (five pain and five no-pain stimuli, presented in random order). Participants in group B performed three blocks: two blocks of face videos (10 trials each, five pain and five no-pain stimuli in random order) interposed by a single block of arm videos (eight trials, four pain and four no-pain stimuli in random order). The interval between blocks was 1 min. Prior to the start of the experiment and after the presentation of instructions, participants completed four practice trials to familiarize them with the task.

Each trial began with the presentation of a red fixation cross in the center of the screen for 500 ms. A video clip was then shown, and the subject was required to rate the target’s pain intensity and his/her own unpleasant reaction on a seven-point scale ranging from “not at all” to “very much.” Subjects were given unlimited time to complete the ratings, but most did so in <1 min. The intertrial interval was 5 s. Each video clip was played once for each subject. Participants’ faces were videotaped by a hidden camera throughout the experiment. After completing the task, participants were required to fill out the Interpersonal Reactivity Index (IRI).

### Physiological measurements and data reduction

Physiological responses to viewing others’ pain, including facial EMG and photoplethysmography (PPG), were measured during the experiment. All physiological signals were acquired and amplified using the respective modules of the Biopac MP150 system and analyzed with AcqKnowledge 4.3 (Biopac Systems Inc.). The sampling rates were fixed at 1000 Hz for EMG and 125 Hz for PPG.

EMG signals from the left CS and ZM muscles were recorded following a standard procedure^58^ with a band-pass filter of 50–500 Hz. Typically, the CS knits the brow into a frown, a sign of negative emotion; the ZM elevates the lip, which commonly occurs in a smile^47^. Raw EMG signals were inspected visually to detect noise and artifacts. Trials (including baseline) with artifacts due to body movement or involuntary facial movement (e.g., pursing the lips or yawning) were excluded from analysis. Data were then integrated, rectified, and segmented. Baseline was determined based on EMG activity during a 2-s presentation of a blank screen (2.5-s pre-stimulus period – .5-s fixation period). The post-stimulus period corresponded to the length of the video clip (0–15 s for pain and 0–7 s for no-pain stimuli). Phasic EMG responses were averaged over the post-stimulus period and expressed as percentage changes from baseline for each participant and muscle.

PPG measures changes in blood volume beneath a photoelectric sensor^59^. A PPG sensor was attached to the participant’s left index finger. PPG activity was converted to pulse rate using an online calculation channel added to the AcqKnowledge software. Pulse rates were also standardized offline using the same procedure as for EMG data.

### Interpersonal Reactivity Index

A Chinese version of the Interpersonal Reactivity Index (C-IRI) was used to measure participants’ trait empathy^60^. The IRI, a 28-item self-administered survey consisting of four subscales (perspective taking [PT], fantasy [FS], empathic concern [EC], and personal distress [PD]), is used widely in the domain of empathy research. The PT subscale assesses the tendency to cognitively imagine another person’s perspective. The FS subscale reflects emotional identification with fictional characters in books, movies, and plays. The EC subscale describes a person’s tendency to have feelings of sympathy and concern for others. The PD subscale measures the extent to which someone feels unease as a result of witnessing another’s emotional distress. The PT and FS subscales assess cognitive components of empathy, and the EC and PD subscales assess other-oriented and self-oriented empathy-related emotional reactions^46^. The IRI was translated into Chinese using back translation, and factor analyses show that the original English and Chinese versions had the same factor components^60^.

### Data analysis

All statistical analyses were carried out using STATISTICA 10.0 software (StatSoft Inc., Tulsa, OK, USA). GraphPad Prism 5.0 (GraphPad Software Inc., La Jolla, CA, USA) was used for graph generation. The significance level was set to *P* < .05 for all analyses.

Demographic variables (age, education) and subjective ratings (attractiveness, pain intensity, unpleasantness, C-IRI scores) were compared between pain targets and no-pain targets, and between groups A and B using Student’s *t*-tests. Gender-based comparisons between groups were conducted using the chi-squared test.

Within-group comparison was also performed. For Group A, ratings and physiological responses (EMG and PPG) to empathy-inducing stimuli (pain *vs*. no pain) were analyzed using Student’s *t*-test. For Group B, two-way repeated measures analysis of variance (ANOVA) was used to examine the effects of condition (pain *vs*. no pain) and stimulus category (arm only *vs*. face only) on observers’ empathic responses. Differences between groups in empathy (including self-reported and physiological data) were assessed using 2 × 2 (group [intact scene *vs*. arm only] × condition [pain *vs*. no pain]) mixed-model ANOVA. Bonferroni comparisons were carried out as *post-hoc* tests. To investigate the relationship between the empathy trait and physiological responses to painful visual stimuli, we conducted Pearson correlation analysis between C-IRI subscale scores and physiological signals (facial EMG and pulse rate). Also, relationship between the physiological measures, as well as between physiological measures and subjective reports of emotional experience, were calculated.

A cluster analysis (K means) was performed to classify observers’ EMG activity depending on similarities in patterns of excitation or inhibition elicited by video stimuli. The percent change from baseline for each participant was used to visualize the temporal distribution of EMG responses. A sliding-window averaging method, in which a 900-ms time window was slid through the entire period of a trial in 100-ms increments, was used to generate the clustering plot. Only > 20% changes from baseline for at least three consecutive windows were taken to represent excitatory or inhibitory responses. The numbers of participants exhibiting excitatory and inhibitory EMG responses to video stimuli were counted and compared between conditions (pain, no pain) and among stimulus categories (intact, face only, arm only) using chi-squared tests.

## Additional Information

**How to cite this article**: Sun, Y.-B. *et al.* Emotional mimicry signals pain empathy as evidenced by facial electromyography. *Sci. Rep.* **5**, 16988; doi: 10.1038/srep16988 (2015).

## Supplementary Material

Supplementary Information

Supplementary Video 1

Supplementary Video 2

Supplementary Video 3

Supplementary Video 4

Supplementary Video 5

Supplementary Video 6

## Figures and Tables

**Table 1:** Demographic characteristics and empathy scores of participants viewing video clips depicting intact and partial scenes.

Variable	Group A (intact scene)	Group B (face-only + arm-only)	*P*-value
*N* (male)	30 (10)	34 (11)	.934
Age (years)	21.00 (2.27)	21.94 (1.67)	.062
Years of education	15.03 (1.65)	15.71 (1.34)	.077
IRI total score	71.90 (8.55)	69.03 (8.38)	.181
Perspective taking	16.60 (2.66)	18.00 (2.52)	.035
Fantasy	18.60 (3.80)	15.12 (4.80)	.002
Empathic concern	20.17 (2.85)	19.53 (3.47)	.429
Personal distress	16.53 (3.52)	16.38 (3.66)	.867

Values are mean (standard deviation) except for *n* (male).

IRI = Interpersonal Reactivity Index.

**Table 2:** Observers’ ratings of others’ pain intensity and their own unpleasantness.

Rating	Group A (*n* = 30)		Group B (*n* = 34)			
	Intact scene		Face only		Arm only	
	Pain	No pain	Pain	No pain	Pain	No pain
Intensity	3.76 (.69)	1.38 (.33)	3.89 (.76)	1.53 (.40)	4.15 (1.04)	1.96 (.74)
Unpleasantness	3.20 (.86)	1.45 (.46)	3.11 (.90)	1.50 (.43)	3.76 (1.15)#	2.11 (.86)

Values are mean (standard deviation).

Pain *vs*. no pain, all *Ps* < .00001.

^#^*P* < .00001 *vs*. face only.
