# Supplementary material for: Emotional mimicry signals pain empathy as evidenced by facial electromyography
Source: Sci Rep. 2015 Dec 9;5:16988. doi: 10.1038/srep16988 (PMC4673531; doi:10.1038/srep16988)
Supplement: Supplementary Information [file srep16988-s1.pdf]

Emotional mimicry signals pain empathy as evidenced by facial electromyography

Ya-Bin Sun[1,2], Yu-Zheng Wang[1,2], Jin-Yan Wang[1]*, Fei Luo[1]

1 Key Laboratory of Mental Health, Institute of Psychology, Chinese Academy of Sciences, 100101, Beijing, China.

2 University of Chinese Academy of Sciences, 100049, Beijing, China.

* Corresponding author

Supplementary Table S1 Demographic characteristics and pain ratings of target subjects showing painful and neutral expressions

**Variable**	**Pain targets (painful expressions)**	**No-pain targets (neutral expressions)**	***P*-value**
Original data (*N* = 51)			
N (male)	19 (7)	32 (8)	.370
Age (years)	21.47 (2.22)	21.63 (2.17)	.812
Self-reported pain (VAS, 0–10)			
Intensity	5.26 (2.24)	3.25 (1.94)	.002
Unpleasantness	3.28 (2.42)	2.56 (1.80)	.226
Data used in the formal experiment (*n* = 20)			
N (male)	10 (4)	10 (4)	1.00
Age (years)	21.90 (2.51)	22.60 (2.41)	.533
Years of education	15.30 (1.34)	16.00 (1.76)	.331
Self-reported pain (VAS, 0–10)			
Intensity	6.01 (2.38)	2.88 (1.88)	.004
Unpleasantness	3.98 (2.69)	2.40 (2.00)	.154
Facial attractiveness (1–7)	3.71 (.38)	3.89 (.28)	.249

Values are mean (standard deviation) except for *n* (male).

VAS = visual analogue scale.

Supplementary Table S2 Between-group comparisons on subjective ratings and physiological measures.

		Condition	Group	Stimuli × group
Intensity	*F*	375.25	10.57	.59
	P	**<.001**	**.002**	.446
Unpleasantness	*F*	300.88	9.53	.26
	P	**<.001**	**.003**	.612
Corrugator supercilii EMG	*F*	15.23	2.08	1.21
	P	**<.001**	.153	.276
Zygomaticus major EMG	*F*	9.90	.22	8.19
	P	**.003**	.640	**.006**
Pulse rate	*F*	5.24	.22	2.53
	P	**.025**	.639	.116

Two-way mixed model analysis of variance, 2 × 2 (condition [pain, no pain] × group [intact scene, arm only]), for self-reported ratings and physiological indices.

EMG = electromyography.

Supplementary Table S3 Numbers (percentages) of observers showing excitatory and inhibitory electromyographic responses

**Response type**	**Group A (*n* = 30)**		**Group B (*n* = 34)**			
	**Intact scene**		**Face only**		**Arm only**	
	Pain	No pain	Pain	No pain	Pain	No pain
Corrugator supercilii						
Excitatory	13 (43%)	4 (13%)	9 (26%)	3 (9%)	15 (44%)	6 (18%)
Inhibitory	0	0	4 (12%)	4 (12%)	5 (15%)	4 (12%)
Zygomaticus major						
Excitatory	12 (40%)	3 (10%)	0	0	0	0
Inhibitory	3 (10%)	3 (10%)	0	0	0	0

Video legends:

Supplementary videos 1-3: Video clips depicting a female participant in pain, who is also the same person as in Fig. 1. The needle penetrated arm scenario (video 2) and the pain expression scenario (video 3), used as stimulus materials for Group B, are derived from the intact painful scenario (video 1) that shows the whole procedure of needle penetration and are applied to Group A.

Supplementary videos 4-6: Video clips depicting a male participant in no-pain condition. The intact no-pain scenario (video 4), used as a control condition for Group A, depicts that the participant receives q-tip touching and shows a neutral expression on his face. The arm no-pain scenario (video 5) in which an arm is touched by another person with a q-tip, and the face no-pain scenario (video 6) in which a face displays a neutral facial expression, are derived from the intact one. Both are employed as the control conditions for Group B.
